# Combinatorial Coding for *Drosophila* Neurons

**DOI:** 10.1371/journal.pbio.0050051

**Published:** 2007-02-06

**Authors:** Rachel Jones

One of the most intriguing questions in biology is how the many different types of cells in an organism can develop from a single cell, the fertilized egg. Nowhere is this question more pertinent than in the nervous system, where each neuron must be endowed with the correct receptors, neurotransmitters, and other crucial molecules to carry out its complex function—animal behavior. The specification of neuronal subtypes relies on the expression of particular combinations of regulatory genes in developing neurons. In a new study, Magnus Baumgardt and colleagues have used the fruit fly *Drosophila melanogaster* to investigate how the expression of specific regulatory genes early in development influences the eventual expression of the correct “combinatorial code” of genes to define cell type.

To address this question, the authors looked at two *Drosophila* neurons that develop from the same ancestor cell, but express different neuropeptides. The “Tv neuron” expresses a peptide called FMRFamide, whereas the “Tvb neuron” expresses Neuropeptide like precursor protein 1 (Nplp1) as well as the dopamine receptor DopR. Interestingly, the authors found that many of the same regulatory genes—including *eya (eyes absent)*, *ap (apterous)*, and *dimm (dimmed)*—are needed for the expression of the correct molecules in both types of neuron. So what determines the difference between them?

Baumgardt and colleagues found that another regulatory gene, called *collier*, or *col*, is crucial. It is expressed in the Tvb neuron (and also in a very similar neuron called dAp) but not in the Tv neuron, making it a good candidate for a role in differentiating between them. When *col* is missing from the Tvb neuron, neither DopR nor Nplp1 is expressed, indicating that *col* is involved in ensuring that the neuron develops correctly.

Mutations in *col* also prevented the expression of other regulatory genes, such as *eya* and *dimm*, which means that *col* probably acts upstream of these genes. Intriguingly, this effect was widespread among other neurons, including the Tv neuron, even though *col* is normally expressed only in the Tvb neuron. This led to the finding that *col* is more widely expressed when these neurons are first generated, but is then rapidly attenuated in all but the Tvb neuron.

**Figure:**
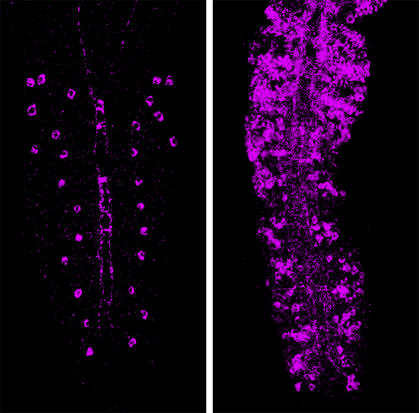
Wild-type Drosophila nerve cord (left) showing expression of the *Nplp1* neuropeptide in a small subset of neurons. Combinatorial misexpression (right) of regulatory genes triggers ectopic *Nplp1* expression in neurons throughout the nervous system.

To investigate the function of *col*, the authors used an ectopic expression system—a method by which a specific gene can be expressed in cells that do not normally express it. When *col* was ectopically expressed in Tv neuronslater in development, when the don't normally contain it, the neurons started to produce Nplp1, and often stopped expressing FMRFamide. This indicates that, late in the development of neurons, *col* causes the Tvb-type neuronal fate, including the expression of Nplp1. But what is its function earlier in the lineage?

The ectopic expression of *col* in all neurons at earlier stages led to strong ectopic expression of the other regulatory genes, including *eya*, in various neurons—in particular, in a part of the developing nervous system called neuroblast row 5, where there was also ectopic activation of Dimm, Nplp1, and DopR. These results suggest that *col* can activate the expression of eya and other regulatory genes in many neurons, but can only cause the generation of Tvb-like neurons, expressing Nplp1 and DopR, in a particular spatial context.

Further experiments using mutations and ectopic expression of *col* and the other regulatory genes showed that *col* acts at several levels during the specification and differentiation of Tvb neurons. In Tv neurons, a similar combinatorial code operates, involving, in particular, the genes *ap*, *dimm*, and *dac (daschund)*. So the two neurons rely on overlapping combinatorial codes of regulatory genes for their development. Misexpression experiments showed that the two overlapping codes seem to be highly specific and potent, able to specify Tvb- and Tv-type neurons throughout the nervous system when ectopically expressed.

These findings shed new light on the ability of combinations of regulatory genes to control the development of different types of cells from the same lineage. They also illustrate how one gene—in this case, *col*—can have different functions at different time points in the developmental process, acting at different levels—first to drive a cell down a subclass-specific path, and then to dictate its unique identity by controlling the expression of its functional markers. Further work will probably reveal other mechanisms that contribute to the regulation of this system. It is also likely that the two main principles revealed by this work—overlapping combinatorial codes of genes, and multiple functions for a single gene at different levels and time points—will be common to many developmental processes, as they allow for extremely high informational value from the use of a limited number of regulatory genes.

